# Analysis of weighted gene co-expression networks and clinical validation identify hub genes and immune cell infiltration in the endometrial cells of patients with recurrent implantation failure

**DOI:** 10.3389/fgene.2024.1292757

**Published:** 2024-04-05

**Authors:** Zhenteng Liu, Shoucui Lai, Qinglan Qu, Xuemei Liu, Wei Zhang, Dongmei Zhao, Shunzhi He, Yuxia Sun, Hongchu Bao

**Affiliations:** ^1^ Department of Reproductive Medicine, Yantai Yuhuangding Hospital Affiliated to Qingdao University, Yantai, Shandong, China; ^2^ Shandong Provincial Key Medical and Health Laboratory of Reproductive Health and Genetics (Yantai Yuhuangding Hospital), Yantai, Shandong, China

**Keywords:** recurrent implantation failure, hub genes, immune cell infiltration, bioinformatics, infertility

## Abstract

**Background:**

About 10% of individuals undergoing *in vitro* fertilization encounter recurrent implantation failure (RIF), which represents a worldwide social and economic concern. Nevertheless, the critical genes and genetic mechanisms underlying RIF are largely unknown.

**Methods:**

We first obtained three comprehensive microarray datasets “GSE58144, GSE103465 and GSE111974”. The differentially expressed genes (DEGs) evaluation, enrichment analysis, as well as efficient weighted gene co-expression network analysis (WGCNA), were employed for distinguishing RIF-linked hub genes, which were tested by RT-qPCR in our 30 independent samples. Next, we studied the topography of infiltration of 22 immune cell subpopulations and the association between hub genes and immune cells in RIF using the CIBERSORT algorithm. *Finally, a novel* ridge plot was utilized to exhibit the potential function of core genes.

**Results:**

The enrichment of GO/KEGG pathways reveals that Herpes simplex virus 1 infection and *Salmonella* infection may have an important role in RIF. After WGCNA, the intersected genes with the previous DEGs were obtained using both variance and association. Notably, the subsequent nine hub genes were finally selected: *ACTL6A, BECN1, SNRPD1, POLR1B, GSK3B, PPP2CA, RBBP7, PLK4, and RFC4,* based on the PPI network and three different algorithms, whose expression patterns were also verified by RT-qPCR. With in-depth analysis, we speculated that key genes mentioned above might be involved in the RIF through disturbing endometrial microflora homeostasis, impairing autophagy, and inhibiting the proliferation of endometrium. Furthermore, the current study revealed the aberrant immune infiltration patterns and emphasized that uterine NK cells (uNK) and CD4^+^ T cells were substantially altered in RIF endometrium. Finally, the ridge plot displayed a clear and crucial association between hub genes and other genes and key pathways.

**Conclusion:**

We first utilized WGCNA to identify the most potential nine hub genes which might be associated with RIF. Meanwhile, this study offers insights into the landscape of immune infiltration status to reveal the underlying immune pathogenesis of RIF. This may be a direction for the next study of RIF etiology. Further studies would be required to investigate the involved mechanisms.

## Introduction

Despite the growing application of *in vitro* fertilization (IVF), success rates have remained relatively constant, and about 10% of patients undergoing IVF treatment experience recurrent implantation failure (RIF), which is a worldwide social and economic concern (Busnelli et al., 2020). The pathophysiologic process of RIF is complicated; thus, there is no consensus guideline for its diagnosis and treatment at present. RIF is frequently defined as failure to achieve a clinical pregnancy after transferring at least four good-quality embryos in at least three fresh or frozen cycles (Coughlan et al., 2014). RIF could be related to embryonic characteristics (male or female origin), embryo transfer, endometrial or immunological factors, or a composite of these. Notably, recent studies highlighted that it has become apparent that endometrial factors have an important role in RIF(Huang et al., 2017; Zhou et al., 2019). Moreover, plenty of literature suggests that endometrial gene expression profiles could be changed in RIF patients (Feng et al., 2018; Ahmadi et al., 2022). Nevertheless, the critical genes and genetic mechanisms underlying RIF are largely unknown.

With the popularity of large-scale gene expression analysis, emerging bioinformatics via public databases can detect differentially expressed genes (DEGs) concurrently at the level of transcription for numerous genes (Koot et al., 2016; Guo et al., 2018; Bastu et al., 2019). However, traditional DEG-based screening methods have the disadvantage of local dataset exploring, so it is quite possible that the master molecules will be missed. Encouragingly, weighted gene co-expression network analysis (WGCNA) can detect co-expression modules and genes in the entire biological system in several samples (Langfelder and Horvath, 2008). By comparable gene expression patterns, genes are grouped to form modules that are studied for their relevance to certain properties, like patient clinical information. Such modules and their main genes can be exploited to find potential biomarkers or therapeutic targets. WGCNA is therefore anticipated to be a novel and potent tool for revealing the RIF potential pathological mechanism.

First, we scanned the microarrays stored in the GEO (Gene Expression Omnibus) database for obtaining genes with differential expression between RIF and healthy fertile controls. Second, functional enrichment analysis was performed to determine potential biological roles and signaling pathways implicated in RIF. Next, functional modules associated with clinical characteristics were scanned employing powerful WGCNA. RIF-associated hub genes were detected and constructed a network of protein-protein interactions (PPI). Concurrently, bioinformatics chip analysis results were validated using quantitative real-time polymerase chain reaction (qRT-PCR). Next, we studied the topography of infiltration of 22 immune cell subpopulations and the association between hub genes and immune cells in RIF using the CIBERSORT algorithm. Finally, diagnostic value, and functional enrichment analyses by Gene Set Enrichment Analysis (GSEA) were sufficiently performed in succession. Thus, our objective was to investigate RIF potential hub genes and molecular mechanism(s), which will expand our understanding of the molecular association between immune infiltration and RIF and propose promising treatment options for RIF patients.

## Materials and methods

### Microarray datasets

**FIGURE 1:**
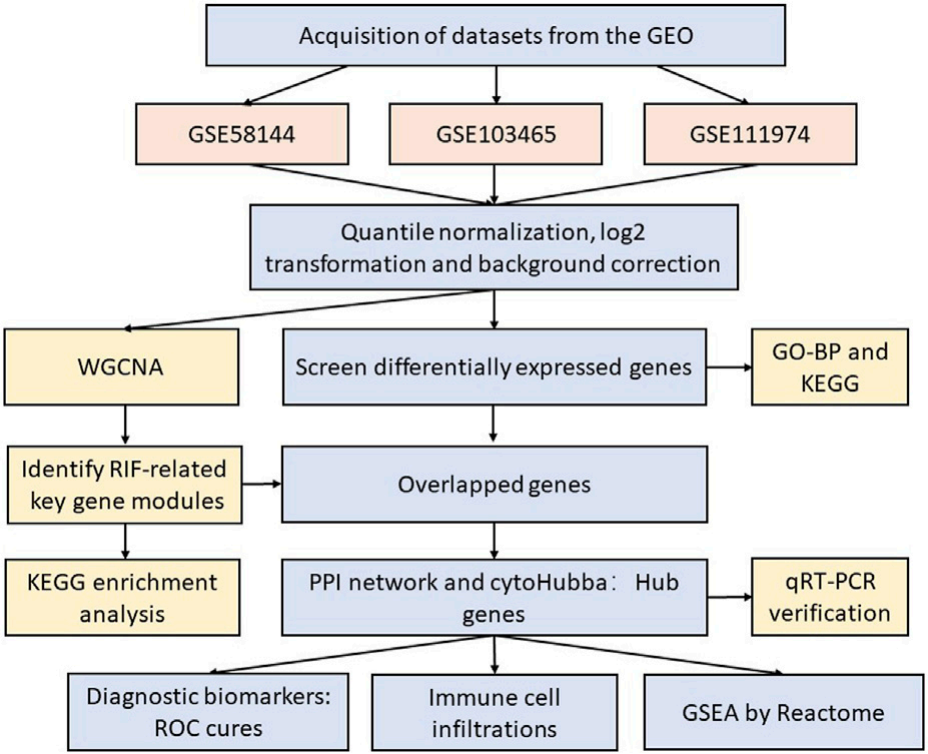
The flow diagram of our study.

Figure 1 displays this research flowchart. The comprehensive profiles of mRNA expression of GSE58144 (Koot et al., 2016), GSE103465 (Guo et al., 2018) and GSE111974 (Bastu et al., 2019) were collected from the GEO (Gene Expression Omnibus) repository (https://www.ncbi.nlm.nih.gov/geo/). All three gene chip profiles were obtained from human mid-luteal phase endometrial biopsies. Dataset GSE58144 was performed according to the platform GPL15789 (A-UMCU-HS44K-2.0), which includes 43 repeated implantation failure (RIF) samples and 72 healthy controls. Series GSE103465 was carried out by GPL16043 (GeneChip^®^ PrimeView^™^ Human Gene Expression Array) and included three RIF samples and three normal control samples. GSE111974, according to the GPL17077 platform (Agilent-039494 SurePrint G3 Human GE v2 8x60K Microarray 039381), included 24 RIF as well as 24 healthy fertile control samples. Supplementary Table S1 provides detailed information about these three datasets.

### Data pre-processing and DEG screening

The raw expression matrix of three datasets was first performed quantile normalization to ensure the columns have the same distribution utilizing the “preprocessCore” package in R tool program (R Foundation for Statistical Computing version 4.1.2). We performed log2-transformation and background correction on GSE58144, GSE103465, and GSE111974 expression profiles utilizing the R package “linear models for microarray data” (limma). Using the annotation file as a basis, probe IDs were converted to gene symbols. For numerous probes mapping to a single gene, the mean expression value of each probe was utilized. The adjusted *p*-value was utilized to regulate the false discovery rate induced by repeated testing. The “limma” program screened DEGs between RIF and fertile control samples. Different datasets have different situations, so it is not feasible to use |log2 fold-change (FC)| > 1 uniformly, which will miss many useful genes. As we use three datasets, we use adjusted *p*-value < 0.05 and |log2 fold-change (FC)|>0 to filter genes and then take the intersection. Moreover, the DEGs were shown as a volcano plot and heatmap using the R packages “ggplot2” as well as “pheatmap.”

### Functional enrichment analysis

Further, DEGs functional enrichment analysis was conducted, which were upregulated or downregulated in at least two datasets by the “VennDiagram” and “RColorBrewer” packages in R. The Gene Ontology-Biological Process (GO-BP) and Kyoto Encyclopedia of Genes and Genomes (KEGG) pathways were then investigated using the “clusterProfiler” and “enrichplot” packages to find potential biological roles.

### WGCNA to identify RIF-related key gene modules

Weighted gene co-expression network analysis (WGCNA) is an algorithm for constructing a co-expression network, which reveals the correlation patterns across genes and provides the biologically functional interpretations of network modules (Yuan et al., 2020). As mentioned before, the intersections of gene lists in GSE58144 were selected to build a co-expression network employing “WGCNA” package (version 1.60). After assessing the existence of apparent outliers through cluster analysis, the one-step network building tool was utilized for co-expression network construction as well as the detection of main modules. Furthermore, to determine the relevance of every module, the module eigengene (ME) was summed according to the initial major element of module expression, and based on the relationship between MEs and clinical features, module-trait associations were evaluated. Then, the correlation strength was assessed using module significance (MS), which refers to the mean absolute gene significance (GS) of each gene inside a module. Relevantly, the GS value was derived by log10-transforming the *p*-value from the linear regression between expression and clinical characteristics. Generally, the modules with the highest MS values were deemed to be the key modules.

### Enrichment analysis of genes in key modules

Using the “clusterprofiler” and “enrichplot” packages, we executed KEGG pathway enrichment analysis to comprehend the biological significance of genes in key modules (Top 20). Moreover, to illustrate the gene-corresponding relationship between the terms, a sub-cluster of terms (the Top 5 in KEGG) was chosen and presented as a network plot (with a similarity of >0.3). Every node depicted a term that has been enriched and colored based on its cluster-ID.

### PPI network construction and hub genes identification

In WGCNA, key genes were determined from key modules with |module membership (MM)|≥ 0.8 and |GS|≥ 0.2. Following overlapping the DEGs and key genes from WGCNA, we inserted these genes into the STRING (http://string-db.org) database to gather target proteins interactions with a medium confidence score of >0.4 [12] and built a PPI network via Cytoscape program (v3.7.2).

Moreover, Hub genes were determined as the central genes for our study employing the Cytoscape plug-in program “cytoHubba” depending on “Closeness,” “Radiality,” as well as “Stress” character calculation.

### Diagnostic effectiveness of hub genes

The functional correlation analysis was conducted among the above hub genes from GSE58144. Moreover, the differentially expressed hub genes were shown regarding adjusted *p*-value and logFC by the heatmap from GSE58144, GSE103465, and GSE111974.

Notably, ROC analysis was conducted to anticipate the biomarkers diagnostic effectiveness. The area under the ROC curve (AUC) value was used to measure the diagnostic effectiveness of GSE58144 dataset in distinguishing RIF from control samples.

### Immune infiltration analysis

The “CIBERSORT” tool in R from GSE58144 was employed to assess the level of immune infiltration in RIF. We investigated the connection between hub genes and immune cell infiltration utilizing correlation analysis as well as the “ggplot2” tool in R.

### Functional correlation and enrichment analyses by GSEA against the Reactome

Applying GSE58144, a functional correlation study was conducted between hub genes and other mRNAs in RIF, and the Pearson correlation coefficient was determined. The top 50 most positively correlated genes with hub genes were chosen for enrichment analysis to indicate the possible function of hub genes. GSEA was conducted on the Reactome pathways utilizing “gsePathway” R tool of the “clusterProfiler.”

### qRT-PCR verification

15 RIF women and 15 healthy fertile controls were recruited between October 2020 and June 2021 in the Yantai Yuhuangding Hospital, a Qingdao University Affiliate. Their mid-luteal phase endometrial tissues were collected to snap-frozen and stored at −80°C until use in the qRT-PCR experiments. Briefly, Trizol (TaKaRa, Dalian, China) was used to isolate total RNA from tissues above the endometrium. The RNA purity was evaluated utilizing a NanoDrop 2000 (Thermo Scientific-USA). cDNA synthesis was performed using the HiScript III RT SuperMix (Vazyme Biotech Co., Ltd., Nangjing, China). The qRT-PCR was conducted with ChamQ universal SYBR qPCR master mix (Vazyme Biotech Co., Ltd., Nangjing, China). Supplementary Table S2 displays the primers (Sangon Biotechnology Company, Shanghai, China) utilized in this work. The 2^−ΔΔCt^ method assessed the relative gene expressions using GAPDH as the reference gene. The complete experimental process was performed for each sample in triplicate. All patients provided informed permission in writing. Ethical approval for the study was granted by the ethical committee of Yantai Yuhuangding Hospital (20221208).

### Statistical analysis

The RT-qPCR results were analyzed by using SPSS (version 24.0, Chicago, IL) and GraphPad Prism (version 8, San Diego, CA) software. Continuous variables were expressed as mean ± standard deviation (SD), and differences between the two groups were compared using Student’s t-test for normally distributed variables. Nonparametric tests were applied for non-normal distribution data. The difference was deemed statistically significant if the *p*-value < 0.05. All microarray data analysis and visualization in this work were conducted with R software (version 4.1.2; https://www.r-project.org/) with the proper packages which are listed in detail in “*Materials and methods*” individually. All the related raw data and R code are provided in the “Supplementary Material”.

## Results

### DEGs identification in patients with RIF and fertile controls

**FIGURE 2:**
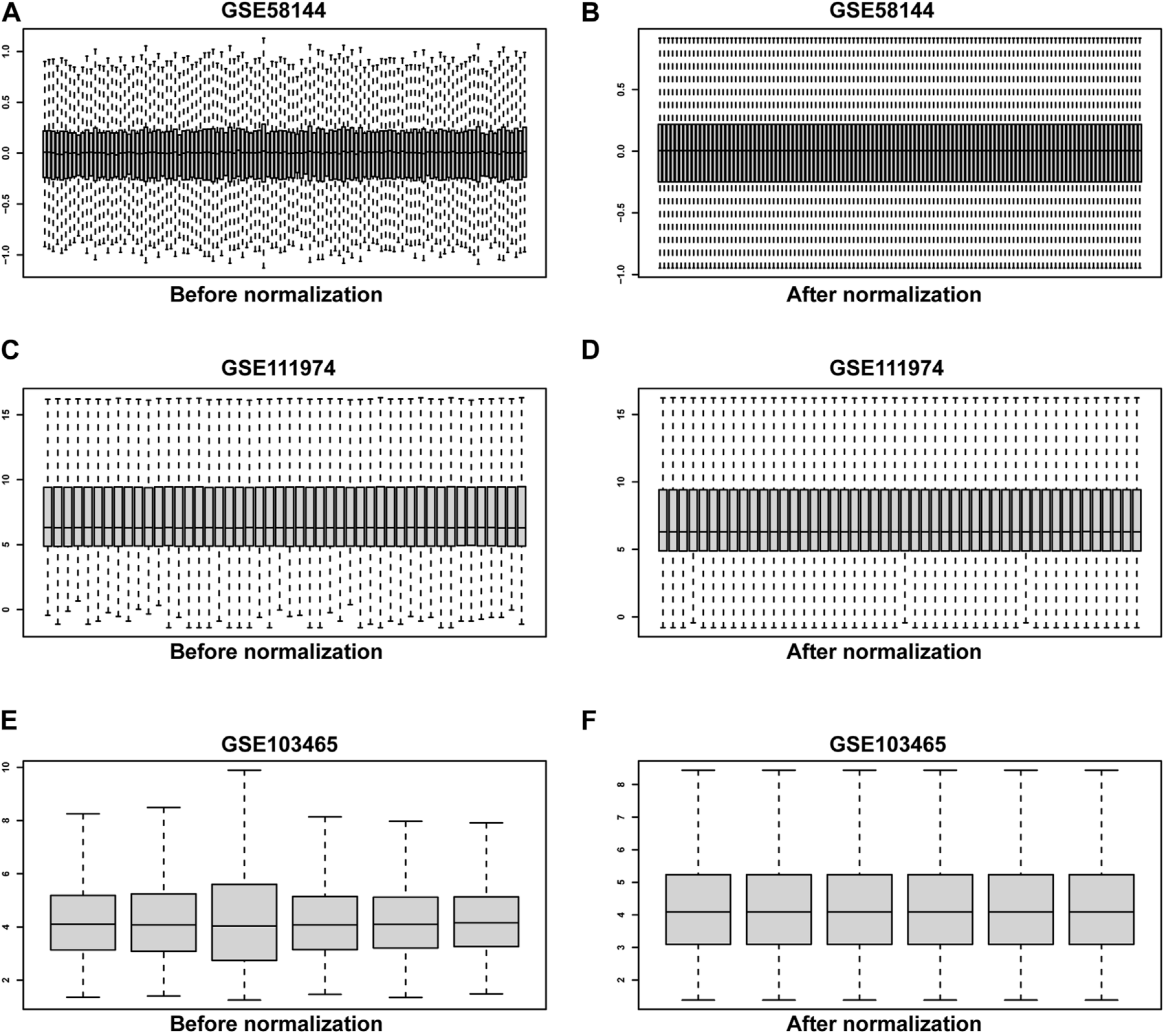
The quantile normalization. Before normalization for GSE58144, GSE111974 and GSE103465 datasets **(A, C, E)**, respectively. After normalization for GSE58144, GSE111974 and GSE103465 datasets **(B, D, F)**, respectively.

**FIGURE 3:**
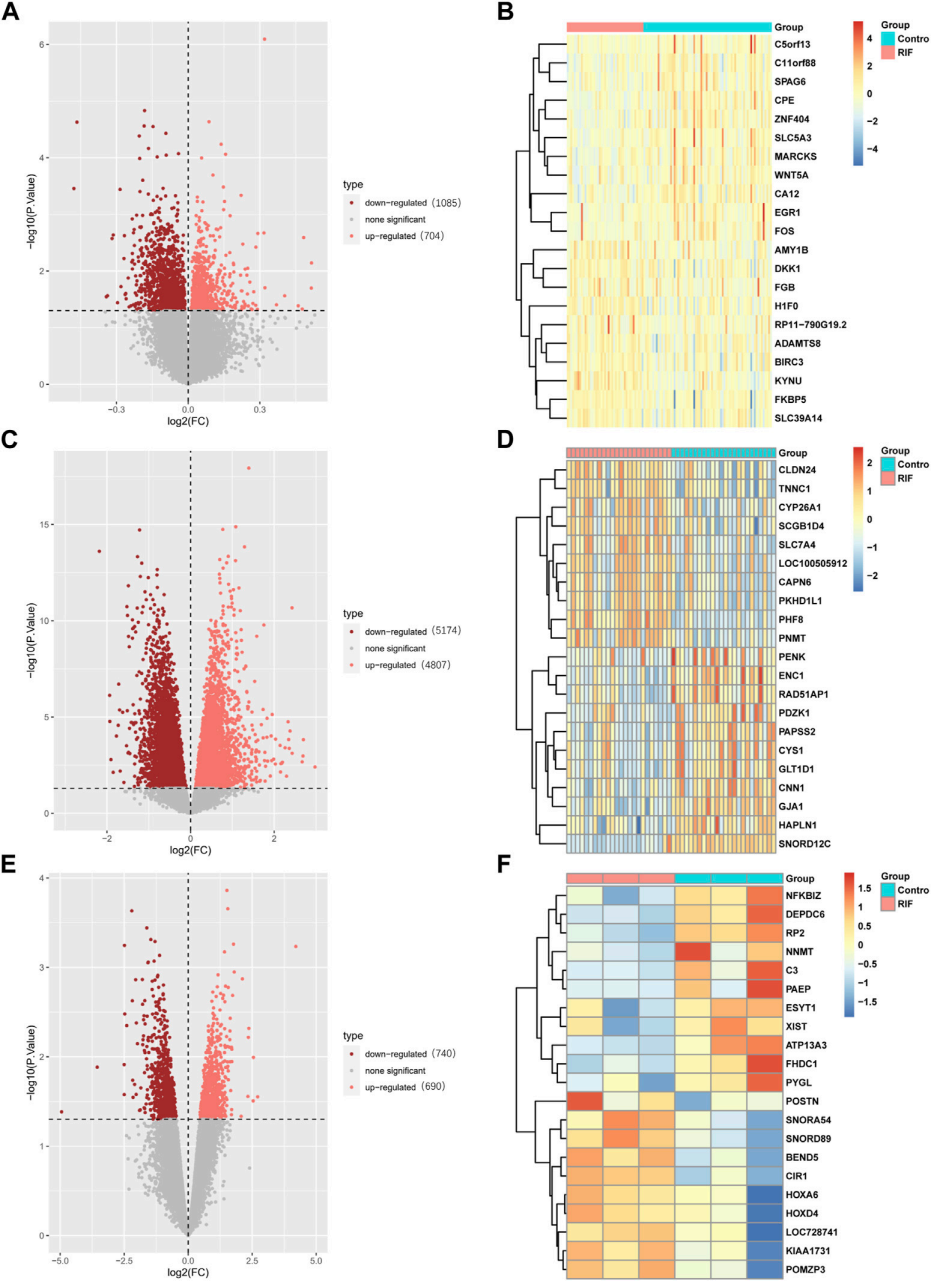
Detection of differentially expressed genes (DEGs) in the GSE58144, GSE111974 and GSE103465 datasets. The expression volcano plots in the GSE58144 **(A)**, GSE111974 **(C)** and GSE103465 **(E)** datasets, respectively. The heatmap of the top 21 DEGs corresponding to the GSE58144 **(B)**, GSE111974 **(D)** and GSE103465 **(F)** datasets.

Three raw microarray datasets, including a total of 99 RIF and 70 normal endometrial tissues, were chosen for the research. Figure 2 displays the data before (A, C, E) and after (B, D, F) quantile normalization, indicating that the normalizing of raw datasets was successful. Following performing data pre-processing on the GSE58144, GSE103465, and GSE111974 datasets, we screened DEGs using the cutoff criteria of adjusted *p*-value <0.05 and |log2 FC| ≥ 0. In GSE58144, we detected 704 upregulated and 1085 downregulated genes. The top 21 DEGs are shown in Figures 3A, B by the volcanic diagram and the expression heatmap. Similarly, a sum of 4807 overexpressed genes and 5174 downregulated genes were identified in GSE111974 (Figures 3C, D). In GSE103465, 690 genes were overexpressed, whereas 740 were downregulated (Figures 3E, F).

**FIGURE 4:**
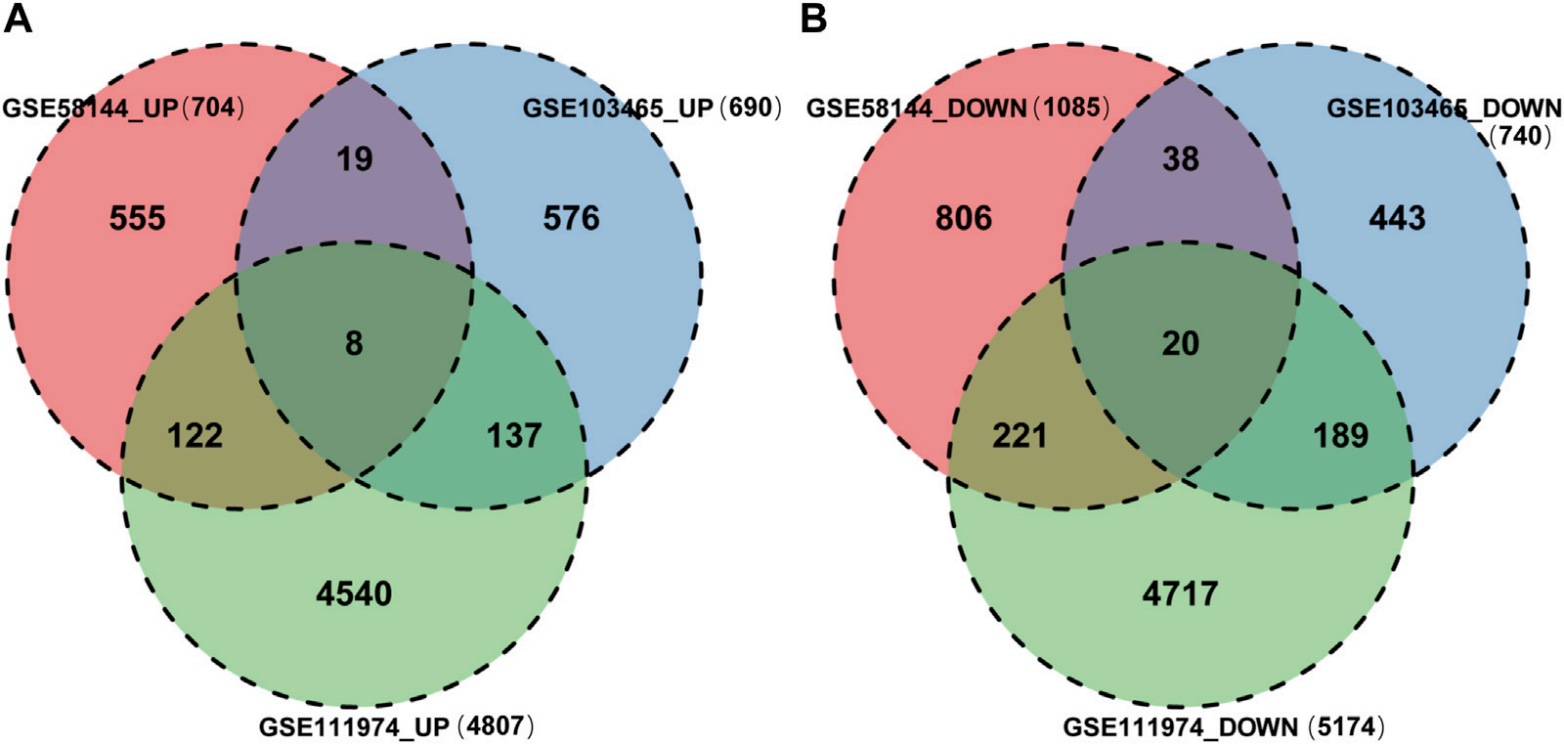
Identification of shared DEGs. **(A)** DEGs upregulated among the GSE58144, GSE111974 and GSE103465 datasets. **(B)** DEGs downregulated among the GSE58144, GSE111974 and GSE103465 datasets.

Finally, 8 upregulated DEGs (Figure 4A) and 20 downregulated DEGs (Figure 4B) were shared among the three datasets, which were displayed through Venn diagram analyses (Figure 4).

### Pathway enrichment analyses

**FIGURE 5:**
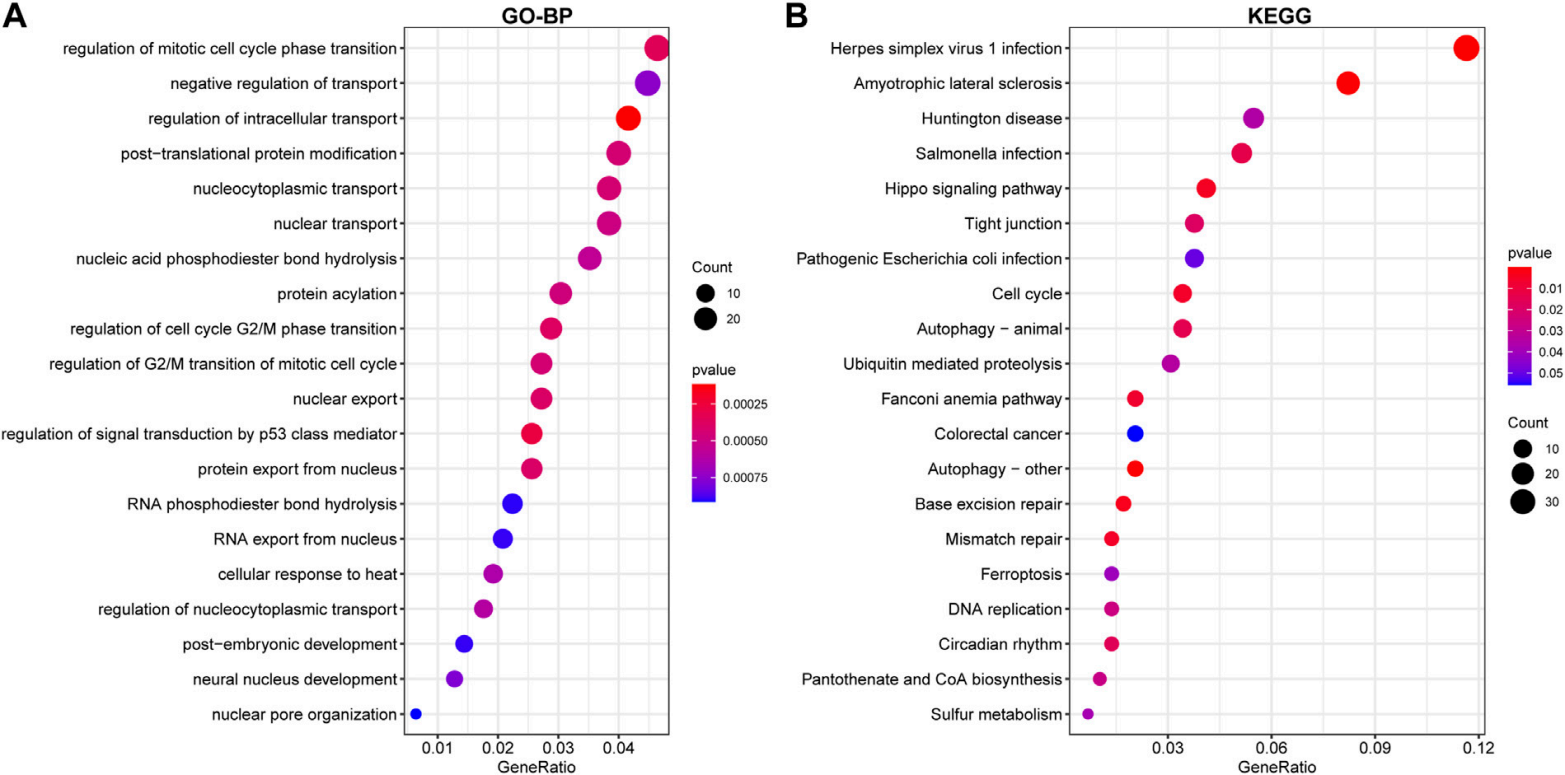
Enrichment of DEGs using Gene Ontology (GO)-BP **(A)** and Kyoto Encyclopedia of Genes and Genomes (KEGG) **(B)** analysis. The larger the circle in the figure, the more genes it contains; lower *p* values are indicated with a stronger red color.

Functional enrichment on the above DEG, which was upregulated or downregulated in at least two datasets, was conducted to discover potential biological roles. GO-BP analysis indicated that RIF was mostly associated with negative control of transport and regulation of mitotic cell cycle phase transition, intracellular transport regulation, post−translational protein modification, nucleocytoplasmic transport, and nuclear transport (Figure 5A). Such DEGs were also enriched in KEGG pathways, including Herpes simplex virus 1 infection, Amyotrophic lateral sclerosis, Huntington’s disease, *Salmonella* infection, Hippo signaling pathway, and Tight junction, which seemed to be associated with RIF (Figure 5B). Supplementary Table S3 demonstrated a list of detailed genes from the datasets that contribute to the identified pathways.

### WGCNA and key modules identification

**FIGURE 6:**
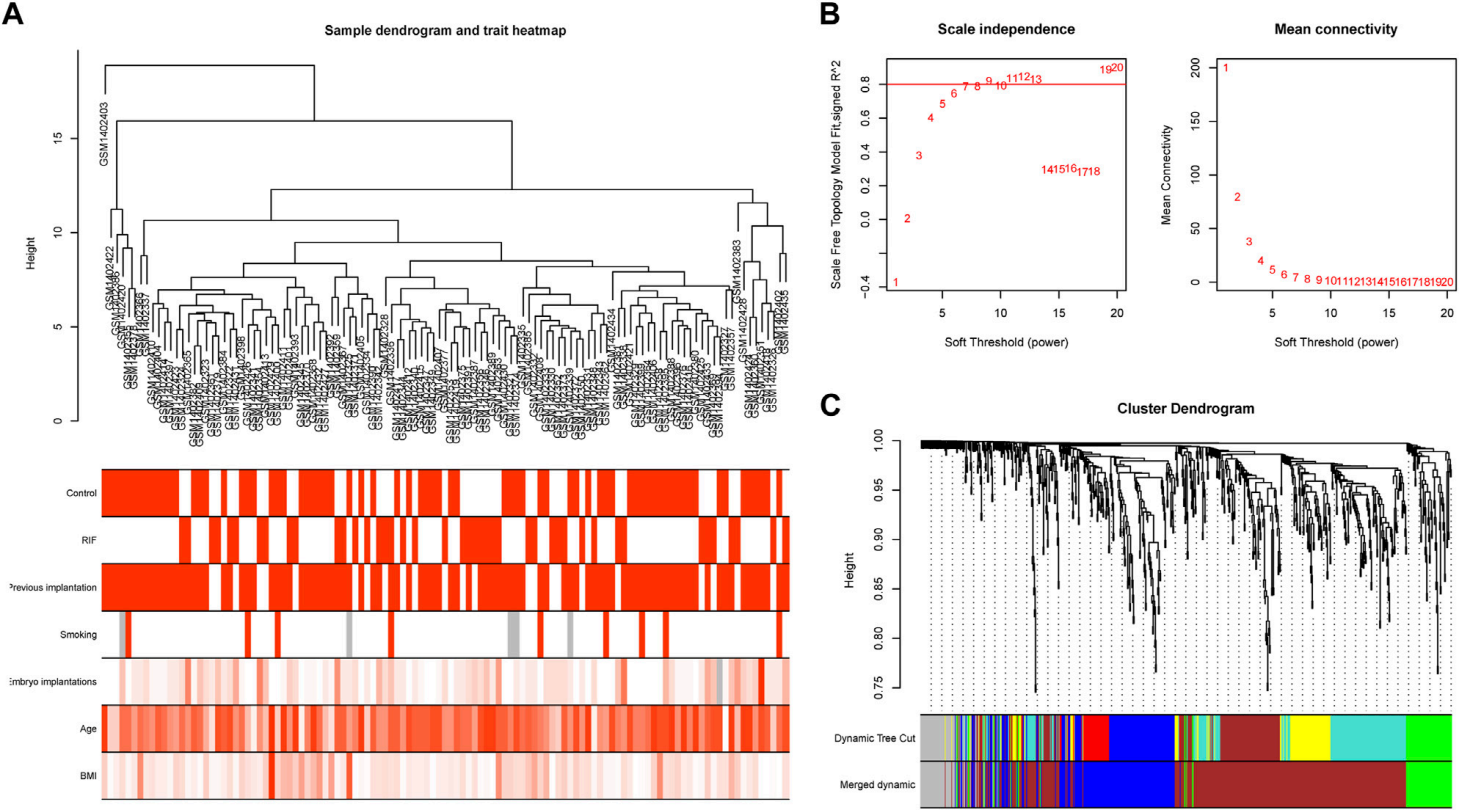
Sample clustering and network construction of the weighted gene co-expression network analysis. **(A)** Clustering dendrogram of 43 RIF and 72 control samples. The color intensity was proportional to disease status (control or RIF) or clinical traits (previous implantation, smoking, embryo implantations, age and BMI). **(B)** Analysis of the scale-free fit index and the mean connectivity for various soft-thresholding powers. The soft-thresholding power of 8 was selected based on the scale-free topology criterion. **(C)** Dendrogram clustered based on a dissimilarity measure (1-TOM). Gene expression similarity is assessed by a pair-wise weighted correlation metric and clustered based on a topological overlap metric into modules. Each color below represents one co-expression module, and every branch stands for one gene.

To study the relation between the aforementioned DEGs and RIF clinical characteristics, we performed a molecular clustering analysis on the GSE58144 dataset using the WGCNA algorithm. On the basis of a dynamic hybrid cut and a scale-free network with topological overlaps, a hierarchical clustering tree was constructed (Figure 6A). The GSE58144 series comprised seven kinds of clinical characteristics: control, RIF, previous implantation, smoking, embryo implantations, age, and BMI. Based on an initial assessment of the data, we determined that a power value of 8 (scale-free R^2^ = 0.82) was an adequate soft threshold for continued investigation (Figure 6B). As a consequence, we acquired four connected modules colored differently: blue (191 genes), brown (405 genes), green (81), and grey (59) (Figure 6C).

**FIGURE 7:**
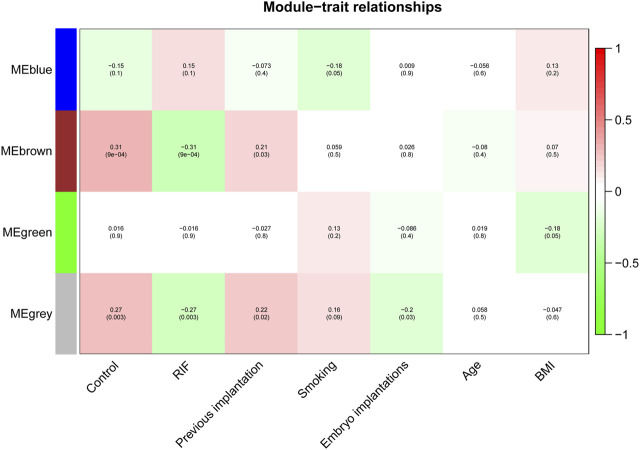
The identification of key modules via weighted gene co-expression network analysis. Heatmap of the correlation between module eigengenes and the clinical traits. The corresponding correlation coefficient along with *p*-value is given in each cell, and each cell is color-coded by correlation according to the color (legend at right).

We investigated the link between the aforementioned four modules as well as clinical parameters to assess the clinical significance of modules. Figure 6 displays that none of the modules had a statistically significant association with prior implantation, smoking, embryo implantations, age, or BMI (*p* > 0.01). In contrast, the brown and grey modules had a high positive association with the control group, but both modules demonstrated a significant negative correlation with RIF (Figure 7). Therefore, subsequent analyses were carried out on genes from the brown and grey modules.

### Enrichment analysis of key modules

**FIGURE 8:**
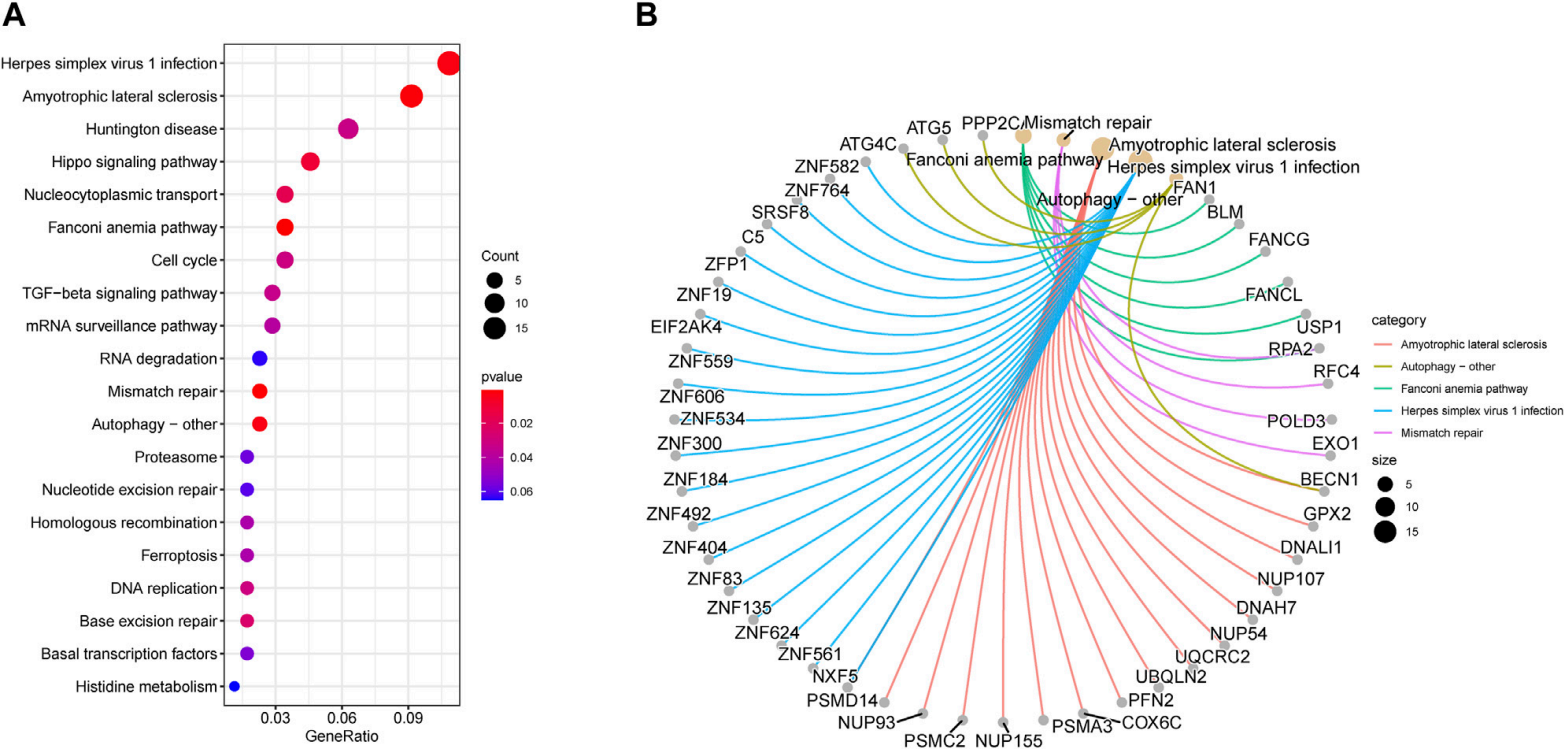
Enrichment analysis of key modules. **(A)** Kyoto Encyclopedia of Genes and Genomes pathway enrichment analysis in brown and grey modules (top 20). The significance of enrichment gradually increases from blue to red, and the size of the dots indicates the number of genes contained in the corresponding pathway. **(B)** Gene-Concept Network: Gene corresponding relationship analysis on the top 5 pathways.

We utilized KEGG analysis for a better understanding of the potential biological role of two key modules related to RIF. Figure 8A displays that KEGG pathway analysis (top 20) indicates that Herpes simplex virus 1 infection and Amyotrophic lateral sclerosis were the highest enriched pathways, followed by Huntington’s disease, Hippo signaling pathway, nucleocytoplasmic transport, as well as Fanconi anemia pathway.

Besides that, we executed gene corresponding relationship analysis between the genes and top 5 pathways (Amyotrophic lateral sclerosis, autophagy–other, Fanconi anemia pathway, Herpes simplex virus 1 infection, and Mismatch repair) enriched by *KEGG* in key modules (Figure 8B).

### PPI network Construction and hub gene identification in the key modules

**FIGURE 9:**
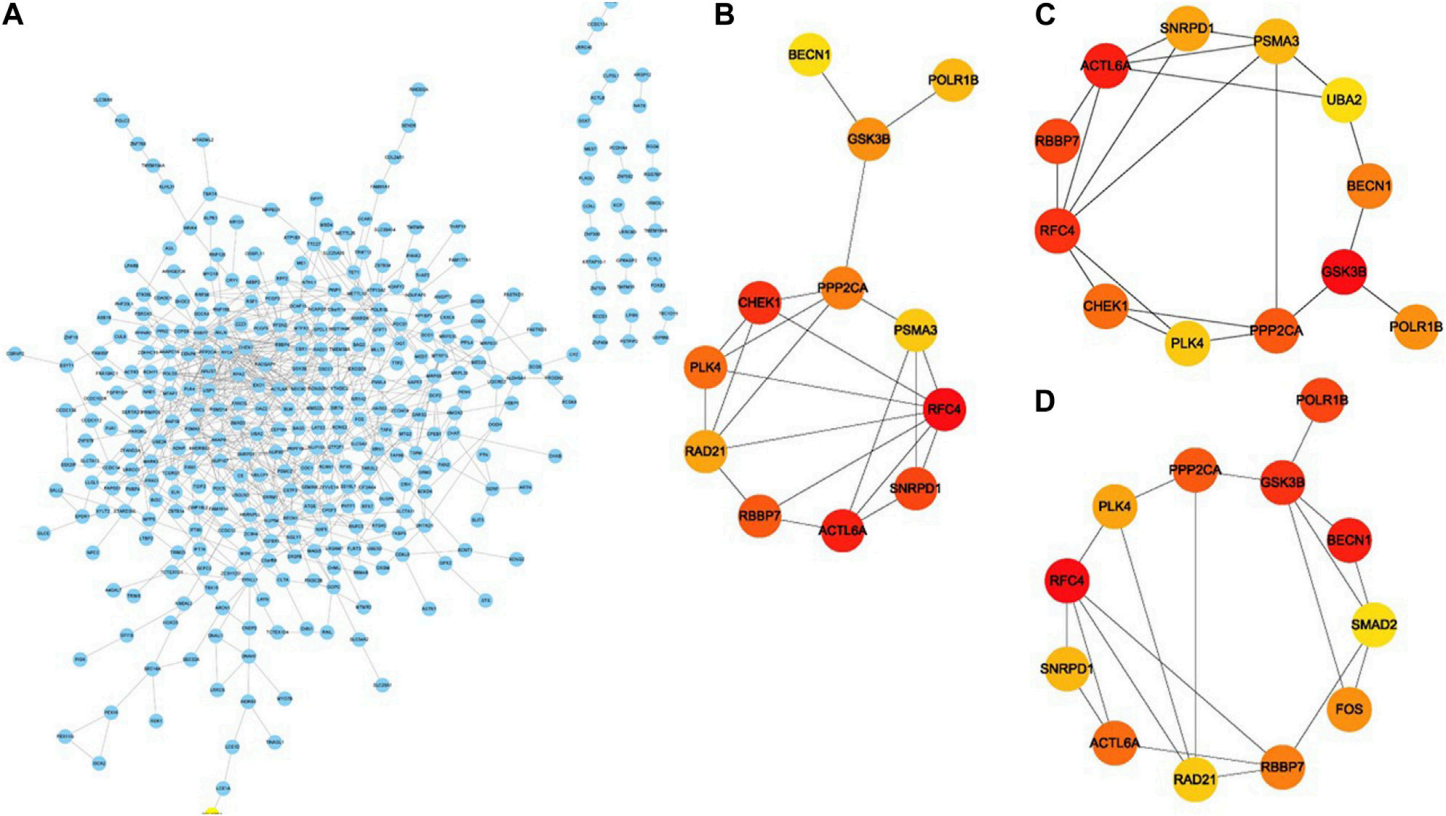
PPI network construction and identification of hub genes. **(A)** The protein–protein interaction network of the overlapped genes. Key genes identified by “cytoHubba” according to three algorithms “Closeness,” “Radiality” and “Stress” **(B–D)**, respectively. The significance of key genes (top 12) gradually increases from yellow to red.

**FIGURE 10:**
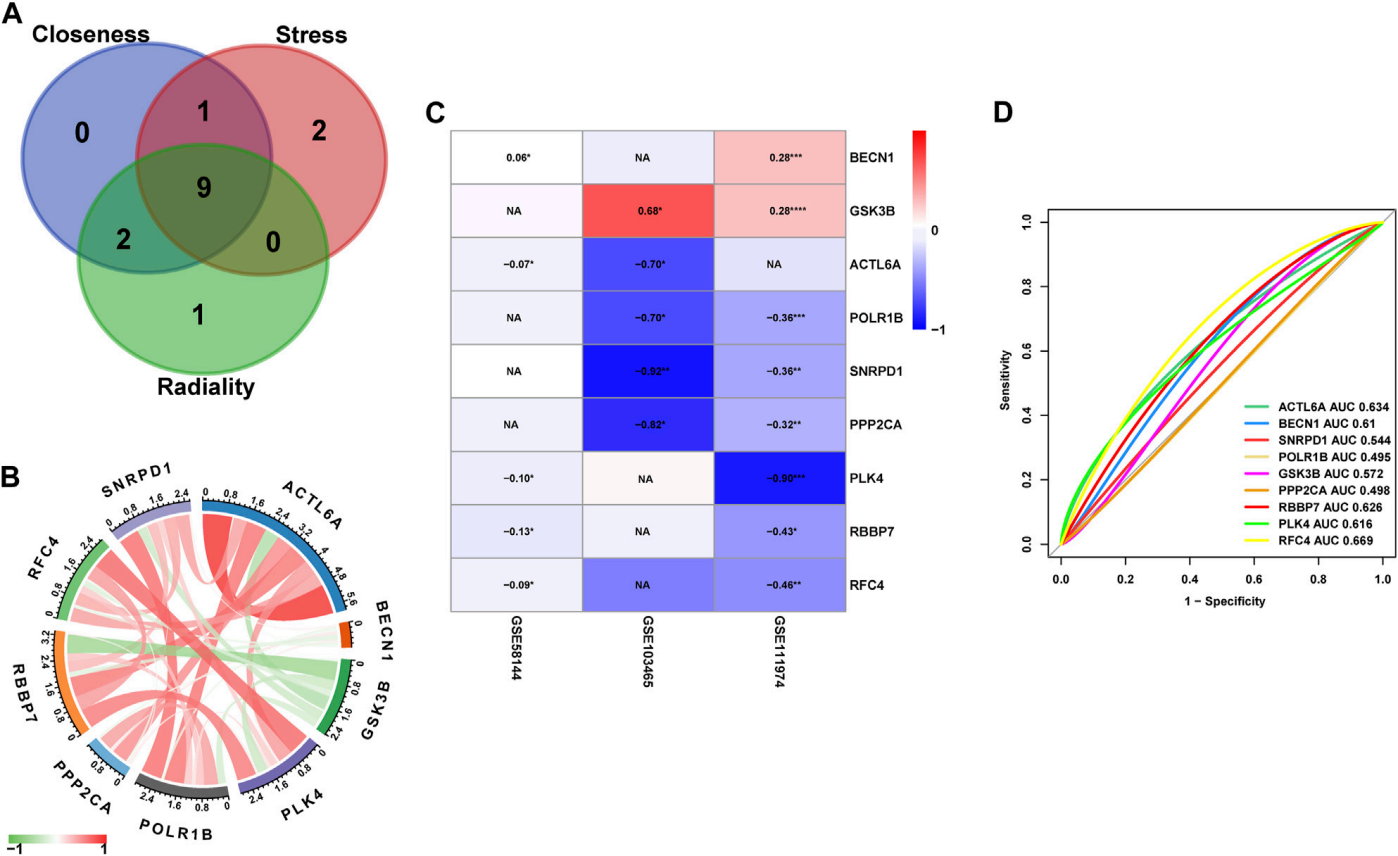
**(A)** The Venn diagram of Hub genes based on three-character calculations from the key modules. **(B)** Correlation analysis between the nine core target genes from GSE58144, where red represents positive correlation, green represents negative correlation, the darker the color, the higher the correlation. **(C)** Heatmap of the nine Hub genes that are differentially expressed in the GSE58144, GSE103465 and GSE111974 datasets. **(D)** The GSE58144 dataset was used to validate the diagnostic effectiveness of the Hub genes for RIF by ROC analysis.

To study in further depth the interaction between genes in the aforementioned brown and grey modules, a PPI network was formed using the STRING database tool, and 464 nodes were displayed using the Cytoscape program (Figure 9A)*.* Further, we used the Cytoscape plug-in “cytoHubba” to screen the hub genes based on their degree of connectivity scores using three algorithms, “Closeness,” “Radiality,” and “Stress” (Figures 9B–D), respectively. After overlapping the above three algorithms by integrating an intersection of genes (top 12), a sum of 9 hub genes were screened out (Figure 10A), which include: actin like 6A (*ACTL6A*), beclin1 (*BECN1*), small nuclear ribonucleoprotein D1 polypeptide (*SNRPD1*), RNA polymerase I subunit B (*POLR1B*), glycogen synthase kinase 3 beta *(GSK3B)*, protein phosphatase 2 catalytic subunit alpha (*PPP2CA*), RB binding protein 7 (*RBBP7*), polo-like kinase 4 (*PLK4*) and replication factor C subunit 4 (*RFC4*). We showed the detailed results of all the above hub genes in Table 1.

**TABLE 1:** Basic information of hub genes.

Name	Ensembl ID	Entrez ID	Description	Location	GSE58144 logFC	GSE103465 logFC	GSE111974 logFC
*ACTL6A*	ENSG00000136518	86	actin like 6A	3q26.33	−0.066958543	−0.700489152	−0.141454232
*BECN1*	ENSG00000126581	8678	beclin1	17q21.31	0.055723909	−0.10473403	0.284900327
*SNRPD1*	ENSG00000167088	6632	small nuclear ribonucleoprotein D1 polypeptide	18q11.2	0.041244512	−0.916550697	−0.360648972
*POLR1B*	ENSG00000125630	84172	RNA polymerase I subunit B	2q14.1	0.001156466	−0.703161264	−0.364147815
*GSK3B*	ENSG00000082701	2932	glycogen synthase kinase 3 beta	3q13.33	0.028225035	0.683436005	0.276002608
*PPP2CA*	ENSG00000113575	5515	protein phosphatase 2 catalytic subunit alpha	5q31.1	−0.0092738	−0.822149207	−0.323529476
*RBBP7*	ENSG00000102054	5931	RB binding protein 7	Xp22.2	−0.127488827	−0.091099307	−0.425808395
*PLK4*	ENSG00000142731	10733	polo like kinase 4	4q28.1	−0.101203559	0.088755112	−0.899178805
*RFC4*	ENSG00000163918	5984	replication factor C subunit 4	3q27.3	−0.085060137	−0.512679937	−0.458995653

### Correlation and expression of hub genes

Correlation analysis of the nine core genes in GSE58144 indicated that: *ACTL6A* expression is positively correlated with *SNRPD1, POLR1B, PPP2CA, RBBP7, PLK4, and RFC4,* and negatively correlated with *GSK3B.* Notably, the gene expression of *GSK3B* exhibited a significant negative correlation to all other genes (Figure 10B).

Moreover, the expression levels of *ACTL6A, BECN1, SNRPD1, POLR1B, GSK3B, PPP2CA, RBBP7, PLK4*, and *RFC4* were presented in the heatmap from all three datasets (Figure 10C)*.* Specifically*, GSK3B* and *BECN1* had significantly enhanced expression in *RIF* in dataset *GSE111974* (*p < 0.05*). However, the levels of expression *of POLR1B, SNRPD1, PPP2CA, PLK4, RBBP7*, and *RFC4* were substantially lower in *RIF* (*p < 0.05*). Compatible with the above findings, the expression of *GSK3B* was also markedly elevated in *RIF* versus control in dataset *GSE103465*, but *SNRPD1* and *PPP2CA* had lower expression in *RIF* (*p < 0.05*)*. BECN1* exhibited higher gene expression in *GSE58144* (*p < 0.05*), while *ACTL6A, PLK4, RBBP7,* and *RFC4* displayed lower expression levels in the *RIF* group.

**FIGURE 11:**
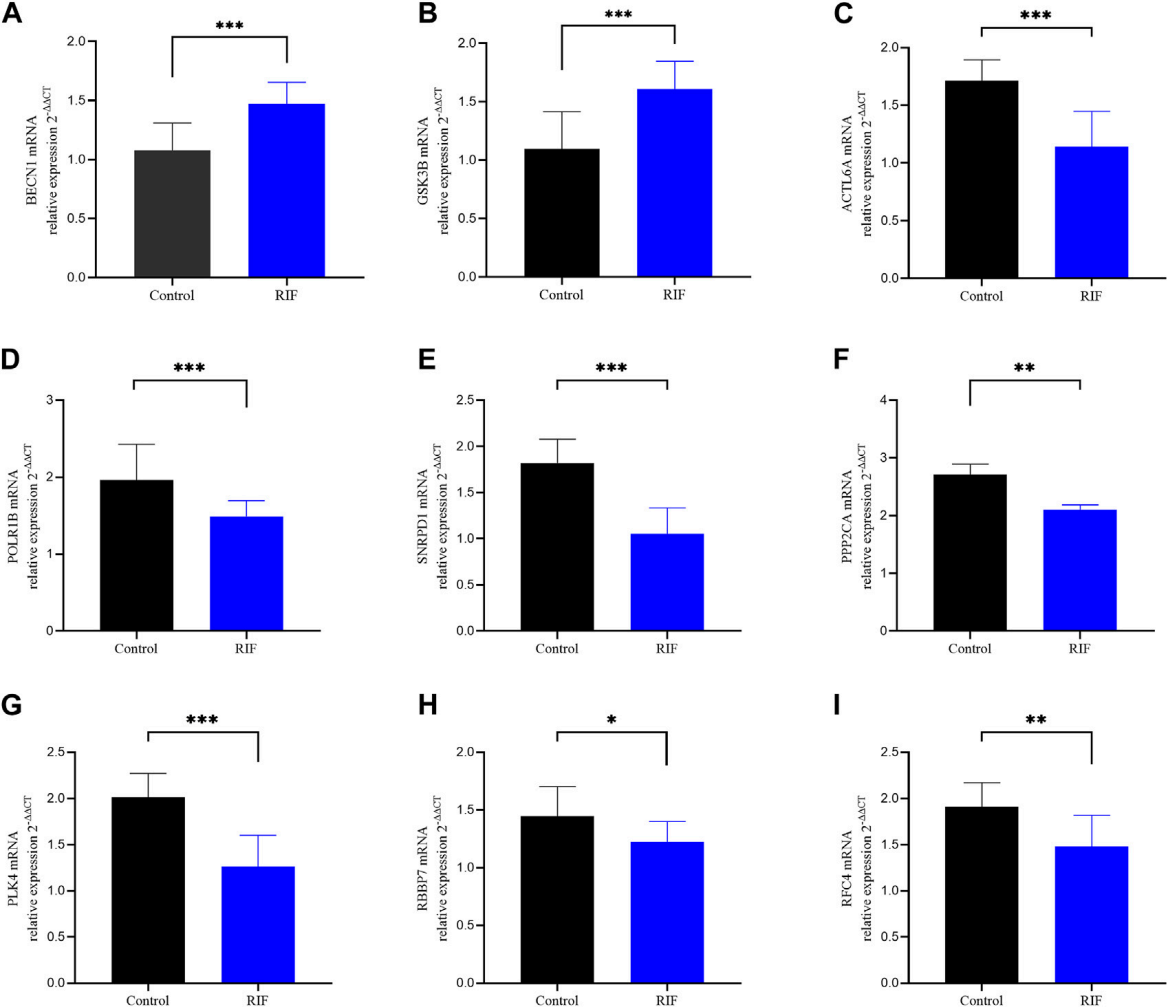
qRT-PCR analysis of the 9 hub genes expression in the indicated groups. Validation of the expression of these 9 hub genes in our study sample. **(A)** BECN1, **(B)** GSK3B, **(C)** ACTL6A, **(D)** POLR1B, **(E)** SNRPD1, **(F)** PPP2CA **(G)**, PLK4 **(H)** RBBP7, **(I)** RFC4.

We further confirmed the expression of nine hub genes via qRT-PCR in our mid-luteal phase endometrial samples. The results were in accordance with the above-described heatmap, i.e., *BECN1* and *GSK3B* were overexpressed in RIF compared to normal endometrial tissues. Meanwhile, *ACTL6A, POLR1B, SNRPD1, PPP2CA, PLK4, RBBP7,* and *RFC4* were significantly lower expressed (Figure 11).

### Diagnostic value of hub genes

*ROC* analysis was employed for the validation of diagnostic effectiveness of the hub genes for *RIF* using the *GSE58144* dataset. Figure 10D depicts the *AUC* values for *RFC4, ACTL6A*, and *RBBP7* were 0.669 (95% CI 0.564–0.763), 0.634 (95% CI 0.529–0.733), and 0.626 (95% CI 0.518–0.729), respectively. Consequently, it appears that these key genes from the *PPI* subgroups do not perform well in the diagnosis of *RIF* (Figure 10D).

### Infiltration of immune cells in RIF

**FIGURE 12:**
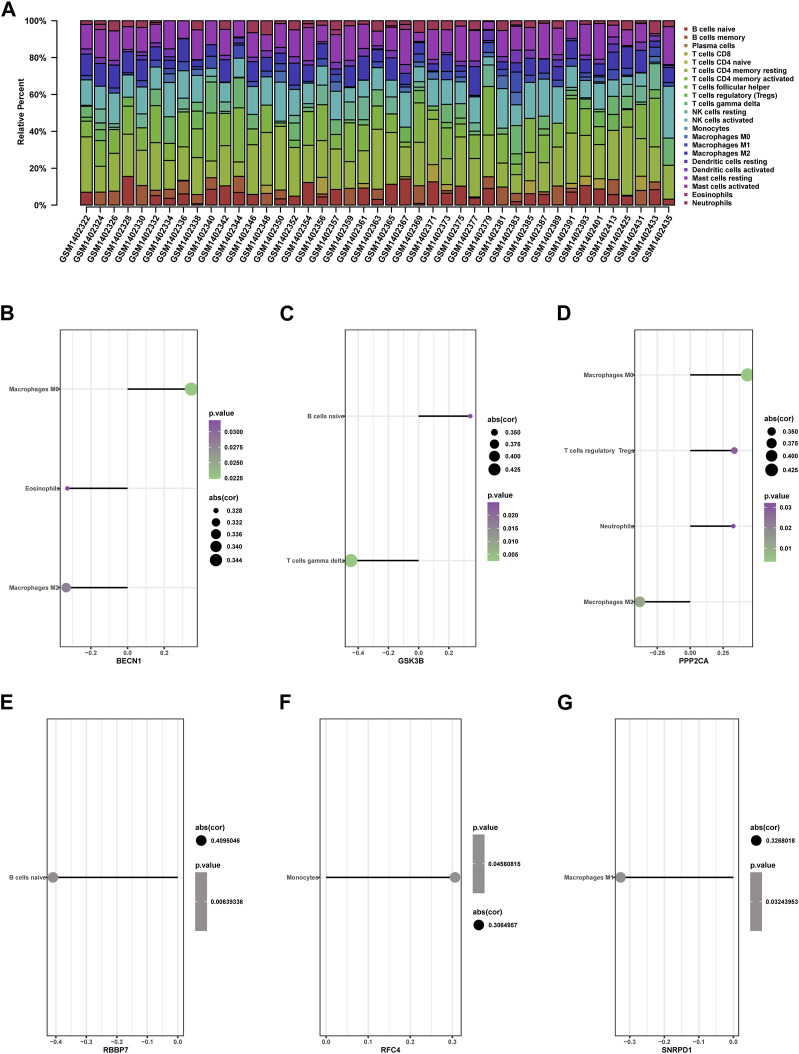
Immune cell infiltration in RIF and Control tissues. **(A)** The composition of 22 kinds of immune cells in each sample was showed in a histogram. **(B–G)** Correlation of the expression of 9 hub genes with the infiltration of immune cells from GSE58144.

Using “cibersoft” package in R, we identified the infiltration landscape of 22 immune cell subpopulations in *RIF* by evaluating the *GSE58144* dataset (7*2 RIF* VS 43 Control)*.* Figure 12A presents that every sample composed of 22 types of immune cells was presented in a histogram. In the histogram, the color indicates the proportion of various immune cells in all samples, and the sum equals 1. Results *i*ndicated that T cells *CD4* memory resting, T cells *CD4* memory activated, *NK* cells stimulated, macrophages *M1*, macrophages *M2,* dendritic cells stimulated, and mast cells resting were the predominant infiltrating immune cells, suggesting that memory *CD4 T* cells and *NK* cells were the two most abundant immune cells in the *RIF* group*.*

### Hub genes Association with differential immune cells

We further investigated the relationship between nine hub genes and immune cell infiltration. Correlation analysis results are presented in the following (Figure 12).

*BECN1* showed positive correlation with macrophages M0 (cor = 0.347636451, *p* = 0.022), but adversely with eosinophils (cor = −0.327840586, *p* = 0.032) and macrophages M2 (cor = −0.334793114, *p* = 0.028) (Figure 12B). GSK3B was positively associated with B cells naive (cor = 0.341707789, *p* = 0.025) and negatively associated with gamma delta T cells (cor = −0.448673168, *p* = 0.003) (Figure 12C). *PPP2CA* presented a positive correlation with macrophages M0 (cor = 0.435657589, *p* = 0.003) and T cells regulatory Tregs (cor = 0.334876257, *p* = 0.028) but adversely with macrophages M2 (cor = −0.382965871, *p* = 0.011) (Figure 12D). *RBBP7* presented a negative association with B cells naive (cor = −0.409504551, *p* = 0.006) (Figure 12E). *RFC4* presented a positive association with monocytes (cor = 0.306495744, *p* = 0.046) (Figure 12F). *SNRPD1* was adversely associated with macrophage M1 (cor = −0.326801828, *p* = 0.032) (Figure 12G).

### The function of nine hub genes in RIF

**FIGURE 13:**
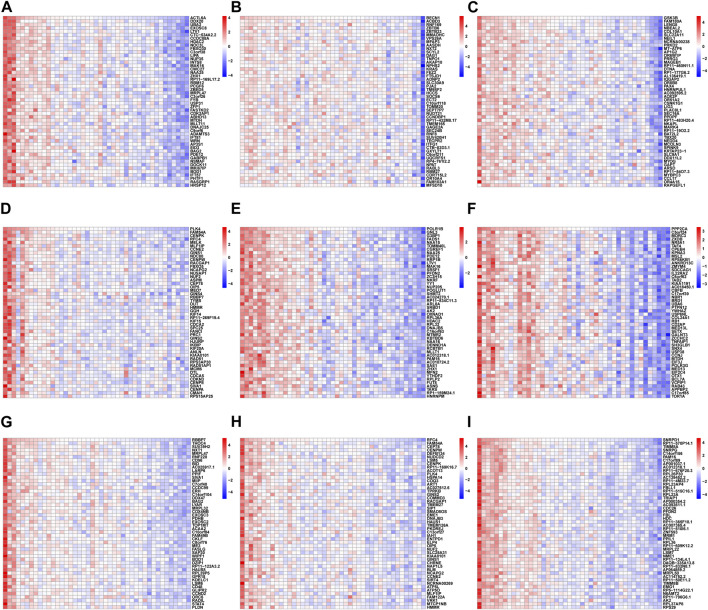
The heatmap of the *correlation analysis* between the 9 hub genes and other all genes in RIF tissues from GSE58144, where red represents positive correlation, blue represents negative correlation; the deeper the color, the stronger the correlation. Each column of the heatmap represents one sample and each row represents one gene. The heatmaps showed the top 50 most positively associated significant genes with **(A)** ACTL6A, **(B)** BECN1, **(C)** GSK3B, **(D)** PLK4, **(E)** POLR1B, **(F)** PPP2CA, **(G)** RBBP7, **(H)** RFC4 and **(I)** SNRPD1, respectively.

To further elucidate the nine hub genes function, correlation analysis between hub genes and all other genes was performed in RIF using the GSE58144 database. As shown in the heatmap (Figures 13A–I), the top 50 most positively associated genes with ACTL6A, BECN1, GSK3B, PLK4, POLR1B, PPP2CA, RBBP7, RFC4, and SNRPD1, respectively, were selected for subsequent enrichment analysis.

**FIGURE 14:**
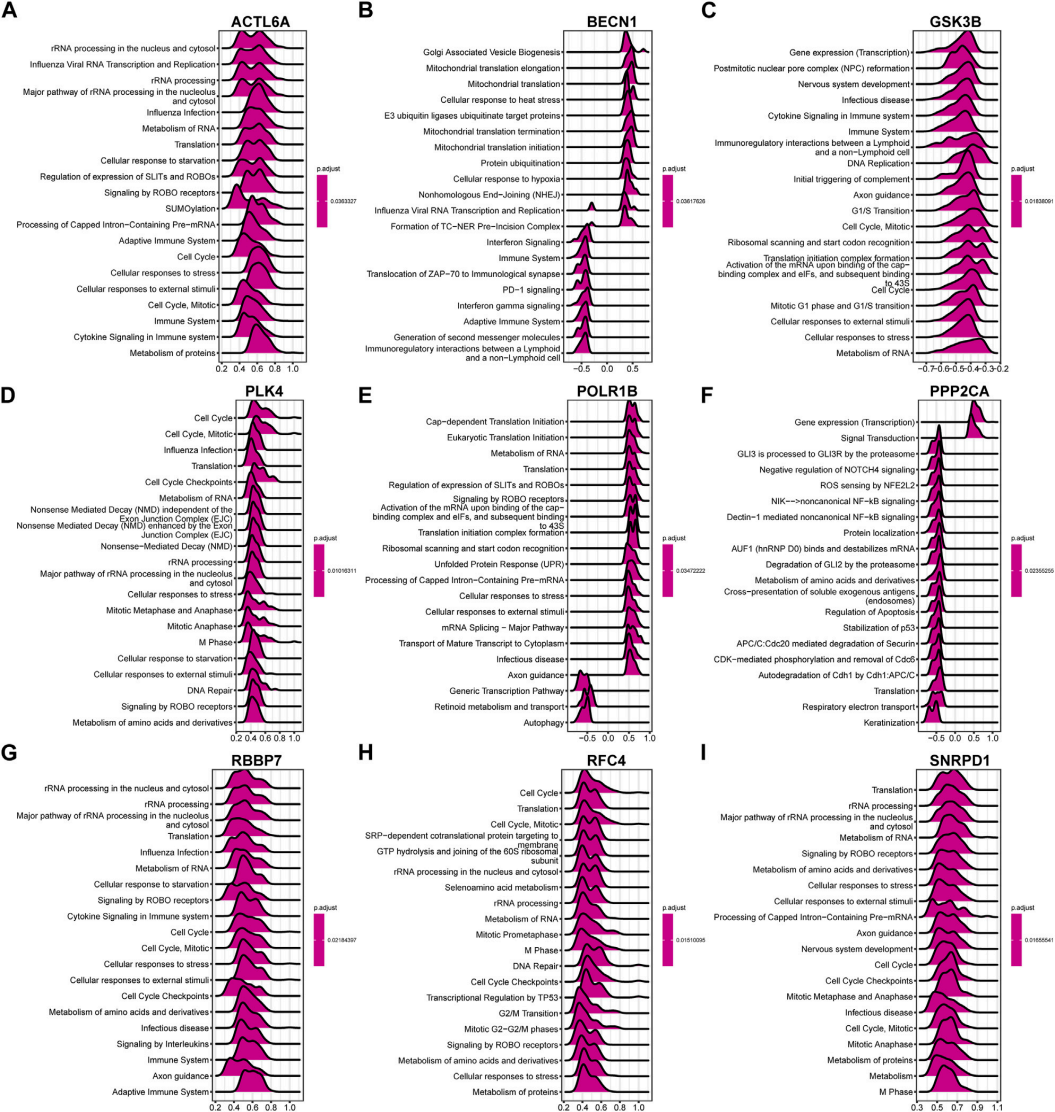
The ridge plot of gene set enrichment analysis (GSEA) against the Reactome pathways (top 20) for the 9 hub genes. Significant GSEA results of the top 50 genes most positively or negatively associated with **(A)** ACTL6A, **(B)** BECN1, **(C)** GSK3B, **(D)** PLK4, **(E)** POLR1B, **(F)** PPP2CA, **(G)** RBBP7, **(H)** RFC4 and **(I)** SNRPD1, respectively.

In accordance with the above correlation analysis findings, the functional pathways enrichment analysis of nine hub genes was conducted using GSEA against the Reactome database. Ridge plot (Figures 14A–I) indicated that the processing of rRNA in the nucleus and cytosol, Influenza Viral RNA transcription and replication, and processing of rRNA were significantly enriched in ACTL6A. The situation of other genes can be easily identified in the ridge plot, which is not shown in detail here.

## Discussion

Up to now, RIF is still not well understood and is an unsolved issue in the field of assisted reproduction, causing significant mental stress, economic strain for families, also several societal issues. Thus, it is vital to examine the potential genetic basis of aberrant endometrial gene expression profiles in patients with RIF to identify better and more accurate biomarkers for diagnosis and treatment.

First, we identified DEGs in the endometrium during the implantation period between RIF and healthy fertile control, which yielded 8 upregulated and 20 downregulated overlapping genes with stable differences in the three-chip datasets. In the further functional exploration of these DEGs, the GO-BP analysis revealed that mitotic cell cycle phase transition, negative control of transport, control of intracellular transport, post−translational protein modification, nucleocytoplasmic transport, and nuclear transport were significantly enriched, which are closely related to the maintenance of body homeostasis. Maintaining cellular homeostasis in the endometrium under oxidative stress is thought to be essential for pregnancy (Qin et al., 2016). A recent study found that downregulation of Sirtuin1 (SIRT1) disrupted the intracellular reactive oxygen species (ROS) homeostasis during the decidualization of human endometrial stromal cells (ESCs) in RIF patients (Li et al., 2021). Although there is no report on the relationship between the above functions and RIF, any imbalance in cellular and molecular endometrial homeostasis may lead to reproductive diseases (Kolanska et al., 2021), raising the possibility that they may cause embryo implantation failure in RIF. This warrants clarification with further studies.

Moreover, the enrichment of KEGG pathways for prevalent DEGs demonstrates that Herpes simplex virus 1 infection and *Salmonella* infection could contribute significantly to RIF. Throughout the human body, microbial communities assemble into distinguishing and stable ecological systems, usually referred to as the “another human genome,” which participates in the internal environment and homeostasis of human body. The female reproductive tract contains different bacterial communities, forming a continuous microbial population from the vagina to the ovary (Chen et al., 2017). It has been shown previously that the abundance of *Lactobacillus* in the vagina has been positively associated with pregnancy outcomes. Compared to individuals who attained clinical pregnancy during the first frozen embryo transfer cycle, patients with unexplained RIF exhibited significantly reduced *Lactobacillus* spp in vaginal canals, and the α-diversity of microbial communities from unexplained RIF was higher than control samples. Moreover, altered Lactobacillus-dominated endometrial microbiota has been reported *to* cause endometritis (Moreno et al., 2016)*,* and some studies suggested an association of chronic endometritis (*CE*) with miscarriage and implantation failure (Cicinelli et al., 2015). A prospective cohort study (*n* = 241) reported that the prevalence of *CE* in the RIF population was up to 33.7% (Kitaya et al., 2017). In combination with the findings of present investigation, we conclude that an imbalance of Lactobacillus-dominated endometrial microbiota can contribute to implantation failure.

Traditional DEG-based screening methods have the disadvantage of locally exploring the dataset, which is most likely to lack master molecules. Encouragingly, WGCNA can effectively detect co-expression modules and genes in the biological system as a whole, which has been widely utilized to discover potential biomarkers or treatment targets. The current result revealed that infection with Herpes simplex virus 1, Amyotrophic lateral sclerosis, and the Hippo signaling pathway were significantly associated with genes in the brown and grey modules, while the genes in the two above modules were a significant negative association with RIF. The Hippo signaling pathway is a signaling mechanism with high conservation that regulates endometrial physiology in a substantial way (Zhu et al., 2017). Previous research uncovered that the upregulation of LATS1 and MOB kinase activator 1A is implicated in RIF patients’ Hippo signaling pathway (Bastu et al., 2019). However, there is no research on the genes related to amyotrophic lateral sclerosis. We hypothesized that dysregulated genes contribute to the progression of RIF via these potential pathways.

According to genes that exhibited a strong association with RIF recognized via WGCNA, the intersected genes with the previous DEGs were obtained with both variance and association. Notably, the nine hub genes listed below were ultimately chosen: *ACTL6A, BECN1, SNRPD1, POLR1B, GSK3B, PPP2CA, RBBP7, PLK4, and RFC4,* based on the PPI network and three algorithms (“Closeness,” “Radiality,” and “Stress”). The levels of expression of the nine hub genes were significantly varied between RIF versus control tissues. Particularly, the expressions of *BECN1 and GSK3B* were significantly elevated, whereas *ACTL6A, POLR1B, SNRPD1, PPP2CA, PLK4, RBBP7, and RFC4* exhibited remarkably decreased expression levels *in RIF* tissues, which were also verified by RT-qPCR in our independent samples containing RIF patients and control. Nevertheless, the diagnostic effectiveness of aforementioned genes for RIF was low. Based on ROC analysis, suggesting on this hand that RIF is a clinical difficulty with heterogeneous etiologic factors and complicated pathogenesis. On the other hand, its diagnosis mainly depends on the special clinical manifestations, and it is currently hard to predict by genetic testing.

*BECN1* and *LC3B* expressions are widely used as autophagic markers. A previous study found that autophagy is drastically enhanced in RIF endometrial tissues and could be implicated in the pathogenesis of RIF(Zhu et al., 2022). Similarly, our result showed that endometrial BECN1 is upregulated in the *RIF* patient compared to normal endometrium. Mehdinejadiani, S. et al. discovered that the *GSK3B* abnormal expression was negatively impacted in the endometrium of women stimulated by clomiphene citrate in comparison with letrozole in polycystic ovary syndrome, primarily liable for the thin endometrium(Mehdinejadiani et al., 2019)*.* This result was consistent with our study, which demonstrated that *GSK3B* had considerably enhanced expression in *RIF.* Besides, *SNRPD1*, one of the key genes encoding core spliceosome constituents, and its elevated protein expression in somatic cells was associated with kidney injury and pulmonary hypertension in systemic lupus erythematosus (*SLE*) patients (Hu et al., 2017). It has been reported that *SNRPD1* could be an oncogene that affects the development of hepatocellular carcinoma by controlling the *mTOR* pathway and autophagy (Wang et al., 2022). Moreover, *PLK4*, an essential member of the polo-like serine-threonine kinase family, is required for centriole duplication regulation. The aberrant expression of *PLK4* resulted in tripolar mitosis and aneuploidy in human preimplantation embryos [25], which led to *RIF*. ACTL6A is an ATP-dependent SWI/SNF regulatory complex protein with chromatin-remodeling components (Mani et al., 2017). Recent studies revealed that follicle-stimulating hormone (FSH)-stimulated glycolysis in ovarian malignancy comprised a higher level of the poor prognostic factor ACTL6A, which forecasted metastasis and prognosis of ovarian cancer patients (Chen et al., 2023). POLR1B, which encodes DNA-directed RNA polymerase I subunit RPA2, has been linked to Treacher Collins and may be involved in cluster headache*s* (Harder et al., 2021)*. PP2A* catalytic subunit *(PP2Aca*) was encoded by the *PPP2CA* gene. A recent investigation revealed that *PPP2CA* variant alleles are significantly correlated with susceptibility to *SLE* (Zhang et al., 2018). RBBP7 is a key element of several complexes for chromatin remodeling and histone modification, that is upregulated in numerous types of cancer and plays contradictory roles in tumors development (Wang et al., 2022). The RFC4 gene, which encodes the fourth biggest subunit of RFC complex, has been found to be dysregulated in a variety of cancers, including head-and-neck squamous cell, hepatocellular, colonic, prostate, and cervical carcinomas (van Dam et al., 2018). However, little relevant studies have been performed to decipher the role of above core genes in endometrial implantation. Hypothetically speaking, key genes mentioned above might be involved in the RIF through impairing autophagy and disturbing proliferation of endometrium, which needs to be further explored.

Embryo quality, embryo endometrium interaction, and endometrial receptivity are essential for successful implantation, and about two-thirds of implantation failures are attributable to insufficient endometrial receptivity (Koler et al., 2009). In particular, well-balanced immune status in the endometrium has been documented as an important determinant of endometrial receptivity. Benefiting from the edge of CIBERSORT to conduct a thorough evaluation of immune cell infiltration, the current study revealed that *CD4* ^*+*^ *T cells* and *NK cells* were the significantly altered cell types in the RIF endometrium. Around the period of embryo implantation, the maternal immune system undergoes significant immunological changes, including enrichment in the different immune cells in peripheral circulation and the uterine microenvironment. These modifications provide an immunologically tolerant environment protecting embryos expressing paternal antigen from maternal antigen-specific T cells and promote successful implantation (Ledee et al., 2016). During embryo implantation, the maternal immune system is comprised of a unique imbalance of subtypes of T lymphocytes (CD4^+^ T cells and killer T cells) which are emerging as a prevalent cause of infertility (Lee et al., 2011). An increasing number of studies showed that in RIF, dysregulation of involved immune cells, including uterine NK (uNK), regulatory T (Tregs), and T-helper cells, have been identified [35-37]. While they interacted during the maternal hemodynamic response to embryo implantation, the changed presence of T cells is also related to lower uNK effectiveness in decidual vessel remodeling (Kieckbusch et al., 2014). Throughout trophoblast invasion, abnormal uNK activity may result in unfavorable effects, including vascular remodeling, local ischemia, and oxidative stress, which are damaging to implantation (Donoghue et al., 2019). Moreover, one report suggested that RIF patients with CE exhibited a substantial overexpression of uterine CD68(+) macrophages, CD83(+) mature dendritic cells, CD8(+) T cells, and Foxp3(+) regulatory T cells, which may contribute to decreased endometrial receptivity and repeated pregnancy failures (Li et al., 2020). Compared to healthy controls, the fraction of CD56^+^ uNK was significantly higher in individuals with RIF, indicating that the intrauterine immunological state of patients with RIF has altered (Von Woon et al., 2022). Consequently, the current findings are in line with earlier reports and demonstrate the significance of these immune cells in RIF pathogenesis, whereas the accurate molecular mechanism needs further research.

Considering the significance of immune infiltration cells and hub genes in RIF, more research reveals that the role of six hub genes (*BECN1*, GSK3B, *PPP2CA, RBBP7, RFC4*, and *SNRPD1*) was considerably associated with immune cells. For example, both *BECN1 and PPP2CA* exhibited a positive correlation with macrophage M0 and a negative correlation with macrophage M2. The BECN1 can promote the autophagy process of macrophages and regulate the immune response of macrophages. A recent study has reported that PPP2CA downregulation improved NF-κB signaling and stimulated macrophage expression of IL-1b, IL-6, and TNF-α (Guo et al., 2020). The outcomes of correlation analysis revealed that hub genes were primarily enhanced in macrophages, eosinophils, B cells, monocytes, and T cells regulatory Tregs, suggesting that the above hub genes may have a function in the incidence and progression of RIF through controlling corresponding immune cells; this hypothesis should be validated in future studies. Last but not least, according to our understanding, this work describes a previously unreported approach through which the ridge plot can display a clear and crucial association between hub genes and other genes and key pathways. This may be a direction for the next study of RIF mechanism.

Compared to previous studies (Wang and Liu, 2020; Dong et al., 2023; Lai et al., 2023), we first used three comprehensive microarray datasets GSE58144 (Koot et al., 2016), GSE103465 (Guo et al., 2018), and GSE111974 (Bastu et al., 2019) comprising 99 RIF and 70 normal endometrial tissues, and both enrichment analysis and WGCNA were conducted to distinguish hub genes associated with RIF. The WGCNA has made the greatest contribution to finding association patterns among genes across samples and interpreting the direct biological role of gene network modules, which makes our results more effective and reliable. In particular, we have validated this study’s results through RT-qPCR in our 30 independent samples. However, some limitations should be acknowledged in the current study. First, since the GEO datasets were exploited retrospectively, no additional clinical information about the patients is available, which could cause some deviations in the analysis of our results. Second, the three datasets come from multiple, heterogeneous studies with different populations, these discrepancies might be related to ethnic differences. Third, although the RNA expression patterns of nine key genes were validated in the present study through laboratory experiments, we did not evaluate their expression profiles on the protein level. Last but not least, exploring the expression profiles of the hub genes in previously published single-cell endometrial datasets could unveil specific cell types or state that play a role in the pathogenesis of RIF. We will collect more endometrial samples and single-cell endometrial datasets for more in-depth study in the future.

## Conclusion

In conclusion, we first utilized WGCNA to identify the most potential hub genes (*ACTL6A, BECN1, SNRPD1, POLR1B, GSK3B, PPP2CA, RBBP7, PLK4, and RFC4*), which might be associated with RIF. Meanwhile, this study offers insights into the landscape of immune infiltration status to reveal the underlying immune pathogenesis of RIF. This may be a direction for the next study of RIF etiology. Further studies would be required to investigate the involved mechanisms.

## Data Availability

The datasets presented in this study can be found in online repositories. The names of the repository/repositories and accession number(s) can be found in the article/Supplementary Material.
