# Supplementary material for: Analysis of weighted gene co-expression networks and clinical validation identify hub genes and immune cell infiltration in the endometrial cells of patients with recurrent implantation failure
Source: Front Genet. 2024 Apr 5;15:1292757. doi: 10.3389/fgene.2024.1292757 (PMC11026622; doi:10.3389/fgene.2024.1292757)
Supplement: Supplementary file 2 [file Table1.DOCX]

Suppl. Table 1. Baseline characteristics of selected datasets.

| Datasets | Platform/ Techniques | Age, mean (RIF *vs* control, years) | Samples size | Timing of biopsy | Country / References | Inclusion Criteria | Exclusion Criteria |
| --- | --- | --- | --- | --- | --- | --- | --- |
| GSE58144 | GPL15789 (A-UMCU-HS44K-2.0) | 34.0 *vs* 34.6 | 43 RIF *vs* 72 control | day LH + 6/7 | Netherlands (Koot et al., 2016) | **Ctrl:** had a history of at least one live birth.  **RIF:** had a history of implantation failure from at least three consecutive IVF attempts (including a total of ≥ 4 good-quality embryos). | 1. tubal obstruction (tubal obstruction factor on hydrosalpinx, salpingitis, etc. were excluded);  2. active pelvic infections, undiagnosed vaginal bleeding, uterine anomalies, endometriosis, karyotype anomalies in one or both partners;  3. unexplained infertility. |
| GSE103465 | GPL16043 (GeneChip^®^ PrimeView^™^ Human Gene Expression Array) | 30.5 *vs* 30.9 | 3 RIF *vs* 3 control | day LH + 7 | China (Guo et al., 2018) |  |  |
| GSE111974 | GPL17077 platform (Agilent-039494 SurePrint G3 Human GE v2 8x60K Microarray 039381) | 31.13 *vs* 32.76 | 24 RIF *vs* 24 control | day LH + 7~10 | Turkey (Bastu et al., 2019) |  |  |
