# Supplementary material for: Analysis of weighted gene co-expression networks and clinical validation identify hub genes and immune cell infiltration in the endometrial cells of patients with recurrent implantation failure
Source: Front Genet. 2024 Apr 5;15:1292757. doi: 10.3389/fgene.2024.1292757 (PMC11026622; doi:10.3389/fgene.2024.1292757)
Supplement: Supplementary file 3 [file Table2.DOCX]

Table S2. mRNA-specific primers of hub genes

| Gene | Primer | Sequence (5'-3') |
| --- | --- | --- |
| GAPDH | Forward | CCGCCTGGAGAAACCTGCCAAG |
|  | Reverse | CACCACCCTGTTGCTGTAGCCG |
| ACTL6A | Forward | GGATGGTTGAAGACTGGGATAG |
|  | Reverse | CCTCCGTTCCACTGTTGTATTA |
| BECN1 | Forward | TCAGGAGGAAGCTCAGTATCA |
|  | Reverse | CCGTAAGGAACAAGTCGGTATC |
| SNRPD1 | Forward | GGTGTGGATGTCAGCATGAATA |
|  | Reverse | TTCCTCTTCCTCTGCCTCTTC |
| POLR1B | Forward | CATACGCTACCTCTCCCATTTC |
|  | Reverse | CCAAACACCAGGCTACTATCTC |
| GSK3B | Forward | AGACGCTCCCTGTGATTTATG |
|  | Reverse | GTAGCCAGAGGTGGATTACTTG |
| PPP2CA | Forward | GTGGCAAATCACCAGATACAAAT |
|  | Reverse | CAAGGCAGTGAGAGGAAGATAG |
| RBBP7 | Forward | GTCACCAGAAGGAAGGCTATG |
|  | Reverse | GAGCCGGTGGCTAGAATAAAT |
| PLK4 | Forward | GGTCAGCCACTCCCAAATAA |
|  | Reverse | CAAACCACTGTTGTACGGTTTC |
| RFC4 | Forward | AACCACCCGATTCTGTCTTATC |
|  | Reverse | GCTGCATGACCCTCATCTATT |
